# Magnetically Induced Current Densities in Zinc Porphyrin Nanoshells

**DOI:** 10.1021/acs.jpca.1c10815

**Published:** 2022-03-18

**Authors:** Atif Mahmood, Maria Dimitrova, Lukas N. Wirz, Dage Sundholm

**Affiliations:** Department of Chemistry, University of Helsinki, P.O. Box 55, A. I. Virtasen aukio 1, FIN-00014 Helsinki, Finland

## Abstract

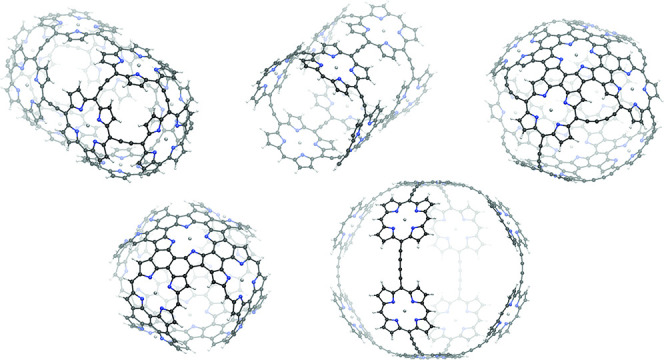

The molecular structures of porphyrinoid cages were obtained by constructing small polyhedral graphs whose vertices have degree-4. The initial structures were then fully optimized at the density functional theory (DFT) level using the generalized gradient approximation. Some of polyhedral vertices were replaced with Zn–porphyrin units and their edges were replaced with ethyne or butadiyne bridges or connected by fusing two neighboring Zn–porphyrin units. Molecule **1** is an ethyne-bridge porphyrinoid nanotube, whose ends are sealed with a Zn–porphyrin. Molecule **2** is the corresponding open porphyrinoid nanotube. Molecule **3** is a clam-like porphyrinoid cage, whose shells consist of fused Zn–porphyrins, and the two halves are connected via butadiyne bridges. Molecule **4** is a cross-belt of fused Zn–porphyrins, and molecule **5** is a cross-belt of Zn–porphyrins connected with butadiyne bridges. The magnetically induced current density of the optimized porphyrinoid cages was calculated for determining the aromatic character, the degree of aromaticity and the current-density pathways. The current-density calculations were performed at the DFT level with the gauge—including magnetically induced currents (GIMIC) method using the B3LYP hybrid functional and def2-SVP basis sets. Calculations of the current densities show that molecule **2** sustains a paratropic ring current around the nanotube, whereas sealing the ends as in molecule **1** leads to an almost nonaromatic nanotube. Fusing porphyrinoids as in molecules **3** and **4** results in complicated current-density pathways that differ from the ones usually appearing in porphyrinoids. The aromatic character of molecules **4** and **5** changes upon oxidation. The neutral molecule **4** is antiaromatic, whereas the dication is nonaromatic. Molecule **5** is nonaromatic, and its dication is aromatic.

## 1 Introduction

The synthesis of porphyrinoid nanostructures with peculiar geometries and potentially interesting properties have gathered interest in the recent years. Porphyrinoid nanorings and nanocages have been obtained by using template-assisted synthesis.^1−7^ Novel porphyrinoid nanostructures have been designed and synthesized by linking the Zn–porphyrins with butadiyne linkers or with larger conjugated bridges.^7−14^ Similar polycyclic aromatic hydrocarbon (PAH) nanostructures^15,16^ as well as planar porphyrinoid nanostructures consisting of arrays or sheets of fused porphyrinoid moieties have also been synthesized.^17−23^ The conjugated nanostructures have appealing topologies with peculiar conjugation pathways, unexpected aromatic character and current-density pathways.^13,24−33^ The synthesized molecules have been spectroscopically characterized with the aim to understand how the aromatic nature changes when increasing the length of the conjugation pathway.^14,34^ The synthesis of polycyclic aromatic hydrocarbon (PAH) nanostructures, porphyrinoid nanorings, porphyrinoid nanobelts, and crossing nanorings of porphyrinoids can also be seen as a generalization of the synthesis of carbon nanostructures.^35,36^

Anderson et al. recently reported the synthesis of porphyrinoid macrorings consisting of Zn–porphyrin units connected with butadiyne linkers forming a nanoring.^10^ Soon after, they managed to synthesize a structure where two porphyrinoid macrorings cross such that they share two Zn–porphyrin units on the opposite sides of the macrorings.^11^ They design novel porphyrinoid nanostructures that are synthesized and spectroscopically characterized with the aim to understand how the aromatic nature changes when increasing the size of the conjugation pathway.^10,11,24,30,32,34,37−39^

These experimental studies inspired us to design and computationally characterize new molecules with a variety of linkers and a different number of macrorings joined into a three-dimensional structure. Here, we investigate a series of novel porphyrinoid nanostructures that, to the best of our knowledge, have not been previously described in the literature. We have studied the effect of having multiple crossing macrorings in the same molecule, as an extension to the experimentally obtained molecules by the Anderson group (molecules **1**, **2**, **3**, and **5**). We further investigated the effect of fusing directly the porphyrin units (molecule **4**).

In the next section, we describe how we constructed the molecules and present the employed computational methods. The results are discussed in detail for each molecule in Section 3. The main results are summarized and concluded in Section 4.

## 2 Computational Methods

The initial molecular structures of the porphyrinoid cages were obtained by constructing small polyhedral graphs where all vertices have degree-4.^40−44^ In molecules **2** and **4**, some edges were removed. The polyhedral graphs were embedded in three dimensions (3D) and preoptimized at a simple force-field level. The vertices were then replaced by Zn–porphyrin units; the edges were replaced with ethyne bridges (molecules **1** and **2**) or butadiyne bridges (molecules **3** and **5**), or two neighboring Zn–porphyrin units were fused (molecules **3** and **4**). The positions where edges have been removed were saturated with hydrogen atoms. The resulting structures were preoptimized with Turbomole at a force-field level and then at the HF-3c level using the minix basis set and imposed symmetry.^45−47^ The final molecular structures were obtained by a subsequent optimization at the density functional theory (DFT) level using the Becke–Perdew functional (BP86),^48−50^ the def2-SVP basis sets,^51^ and the D3(BJ) semiempirical dispersion correction.^52,53^

Nuclear magnetic resonance (NMR) shielding tensors were calculated at the DFT level using the B3LYP functional and def2-SVP basis sets.^29,54−56^ The density matrix and the magnetically perturbed density matrices obtained in the NMR shielding calculations as well as basis-set and structural data were used as input for the calculations of the magnetically induced current density using the gauge-including magnetically induced current method (GIMIC).^57−60^ GIMIC is a freely available program interfaced to common quantum chemistry software packages including Turbomole that we employed in this study.^61^

The aromatic character of the porphyrinoid cages was assessed by investigating the current-density pathways induced by an external magnetic field. The magnetically induced current density is a unique property of the molecular electronic structure.^60,62^ Applying a magnetic field in a chosen direction gives rise to the induced current-density vector field consisting of multiple vortices and domains embedded in each other. The direction of the flux in each vortex can be assigned as positive when it flows in a clockwise direction when looking toward the negative direction of the magnetic field vector, and negative when the flux follows the opposite direction. Tropicity is a global property of the current-density field that can be determined only by following a streamline around the entire current-density vortex. Vortices with positive current-density flux are called diatropic, while those with negative flux are paratropic. Classical electrodynamics can only describe diatropic current density, whereas paratropic contributions are a purely quantum-mechanical effect. The degree of aromaticity can be estimated by integrating the current-density flux associated with a molecular ring.^57^ Molecules sustaining a strong diatropic current density are aromatic, whereas strong paratropic current density is associated with antiaromatic character.

The strength of the current density can be obtained by placing a plane that intersects a given chemical bond and by integrating the current-density flux passing through the plane. We calculate the current density in discrete points on the plane and integrate numerically the strength of the current-density flux. Integration planes are placed through chemical bonds in order to avoid the strong current-density domains arising from the core and the valence shells of atoms, which may introduce inaccuracies. The current density can also be investigated visually by means of streamlines as implemented in the Paraview program.^63^ A set of points in a sphere with a certain radius and point density are employed to trace the vector field using the Runge–Kutta method.^64,65^

## 3 Results and Discussion

### 3.1 Molecule **1**

**Figure 1:**
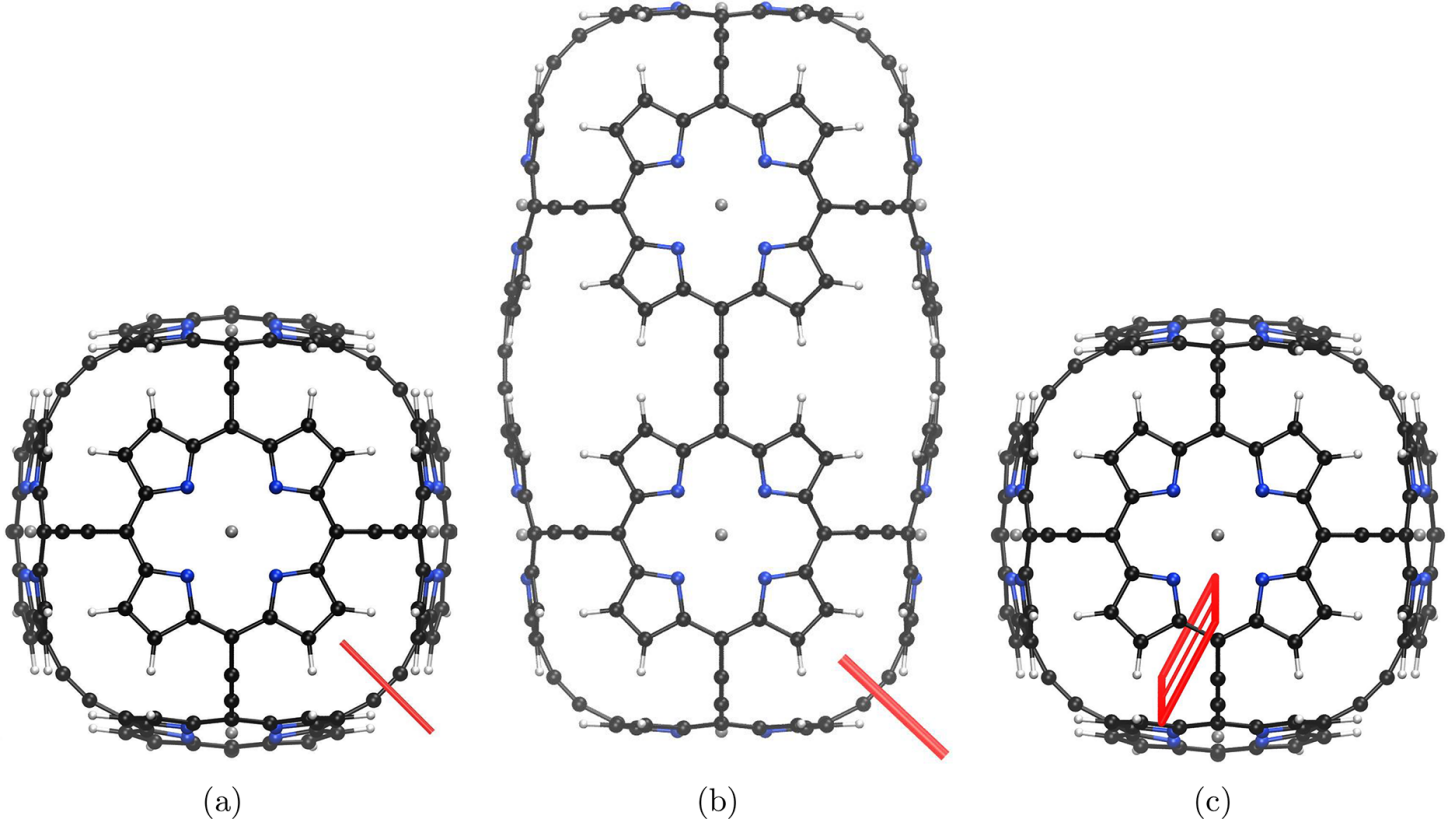
Molecule **1** is shown from different perspectives. The red bars illustrate integration planes used in the current density analysis. Planes 1A, 1B, and 1C are shown in parts a–c, respectively. The magnetic field points toward the viewer.

**Figure 2:**
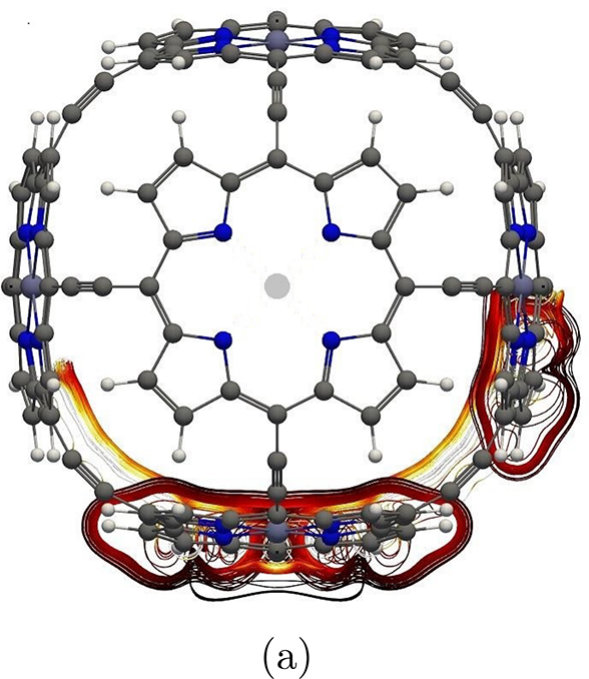
Global paratropic ring current and the local pathways associated with the Zn–porphyrin unit parallel to the magnetic field in molecule **1**. The color scale represents the strength of the current density in 3D, such that white is the strongest and black is the weakest.

Molecule **1** consists of 10 Zn–porphyrin units, which are all interconnected via ethyne bridges at each of their *meso* carbon atom. Thus, the molecule can be described as three crossing macrorings—one comprising six Zn–porphyrin units and two macrorings consisting of four Zn–porphyrin units each perpendicular to the bigger ring. In the current-density analysis, integration planes were positioned such that they cut a linker of the bigger macroring (plane 1B), one of the smaller macrorings (plane 1A), or the *meso* bond of one of the Zn–porphyrin units (plane 1C). The magnetic field is oriented perpendicularly to the investigated molecular ring. The integration planes are visualized in Figure 1. The integrated strength of the current density of −19.5 nA·T^–1^ obtained using plane 1B, which crosses the big macroring, suggests that the molecule is antiaromatic. For reference, the ring-current strength of benzene is 12 nA·T^–1^ at the same level of theory.^66^ There are two major current-density pathways. On the outside, there is a weakly diatropic global ring current, whereas on the inner surface of the molecular structure, there is a strong paratropic ring current. The current density of molecule **1** is illustrated with streamlines in Figure 2. The current density forms long pathways tracing the whole perimeter of the macrorings that are perpendicular to the magnetic field. The Zn–porphyrin units oriented in a parallel direction to the field only sustain local current-density vortices, which is typical and expected.

The integrated values given in Table 1 show that the ring current of −26.2 nA·T^–1^ in the smaller macrorings is stronger than in the bigger one. Given that there are two identical rings, this means that for the molecule as a whole in this direction of the magnetic field, the total paratropic current strength is −52.4 nA·T^–1^. Its magnitude might be overestimated at the B3LYP level.^26^ However, the calculations suggest that the molecule is strongly antiaromatic.

**Table 1:** Strength of the Diatropic and Paratropic Contributions to the Net Strength of the Current Density (in nA·T^–1^) Passing Different Integration Planes in Molecule **1**

plane	diatropic	paratropic	net
1A	1.2	–27.4	–26.2
1B	2.1	–21.6	–19.5
1C	20.0	–5.1	14.9

Integration of the current density reveals that the Zn–porphyrin unit perpendicular to the magnetic field in Figure 1c is 14.9 nA·T^–1^, whereas each Zn–porphyrin unit perpendicular to the plane seen by viewer in Figure 1a sustains a local ring-current of 15.4 nA·T^–1^. The ring currents of the Zn–porphyrin moieties are about 50% weaker than the ring currents of free-base porphyrin and Zn–porphyrin molecule, whose ring-current strengths are about 27 nA/T.^59,67^ The Zn atom sustains an atomic current of 67.7 nA/T. The net ring-current strength induced by a magnetic field parallel to the bigger diameter of the ellipsoid-shaped molecular structure includes twice the strength of the ring current of the Zn–porphyrin since there are two of them, i.e., one on each of the poles of the ellipsoid and twice the ring-current strength from plane 1A since there are two small macrorings. The net strength becomes −22.6 nA·T^–1^, which means that the molecule is globally antiaromatic for all directions of the external magnetic field.

### 3.2 Molecule **2**

**Figure 3:**
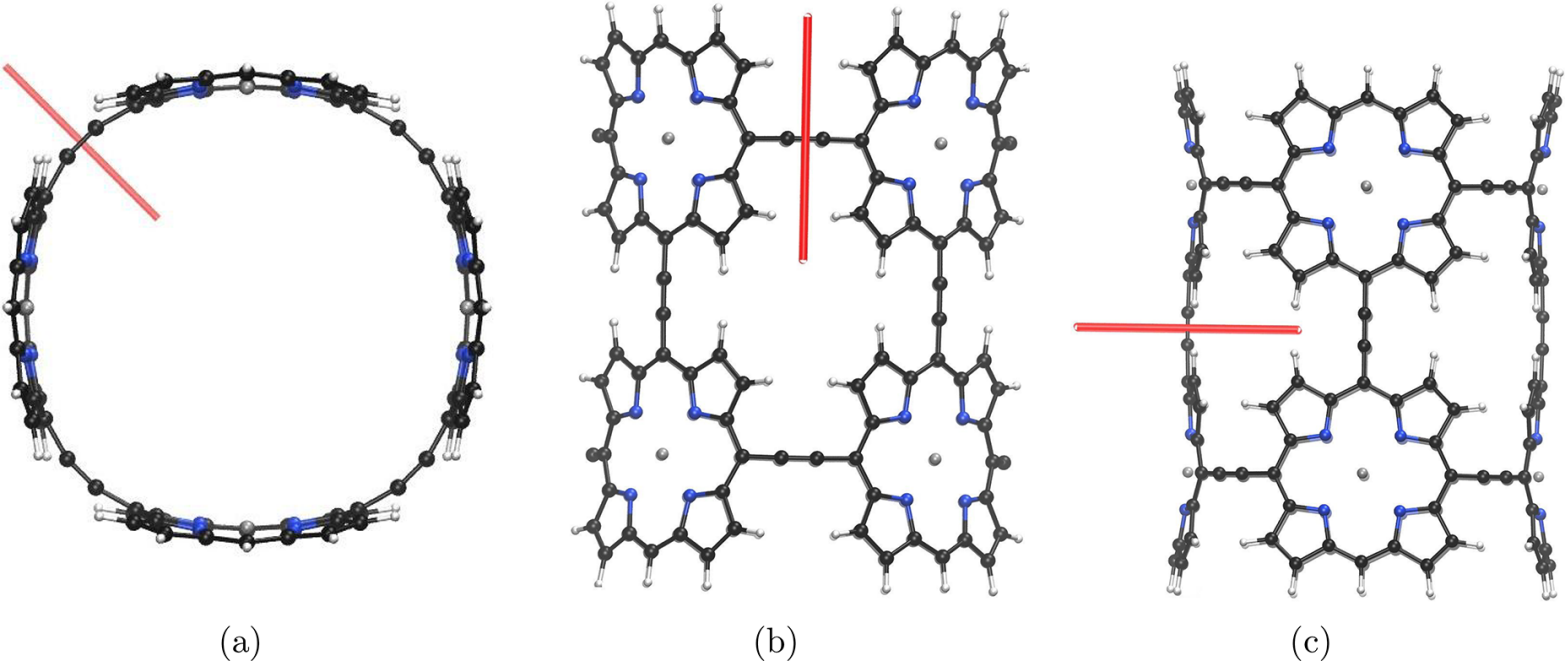
Red bars illustrating integration planes in molecule **2** used in the current density analysis. Planes 2A, 2B, and 2C are shown in parts a–c, respectively. The magnetic field points toward the viewer.

**Figure 4:**
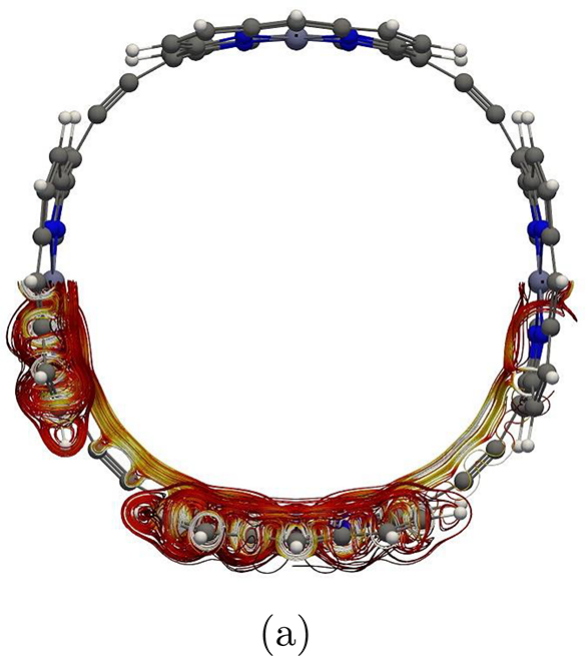
Illustration of the global paratropic current in molecule **2**.

Molecule **2** consists of eight Zn–porphyrin units connected by ethyne linkers. It resembles molecule **1**. However, the two Zn–porphyrin units at the poles of molecule **1** are missing in the tubular molecule. Hence, there are no macrorings consisting of six Zn–porphyrin units as in molecule **1**. It can also be described as two macrorings of four Zn–porphyrin units parallelly linked to each other. Due to symmetry, the same integration plane was employed in two of the calculations; however, the magnetic field direction was different, pointing toward the viewer as illustrated in Figure 3. The strength and the tropicity of the current density show that molecule **2** is antiaromatic. The strongest ring current was obtained by integrating along plane 2A where the magnetic field is perpendicular to the macroring. However, the molecule consists of two such macrorings; thus, in this alignment of the field, the magnetically induced current density is actually twice as big or −35.8 nA·T^–1^, which is about 70% of the strength of the corresponding ring current in molecule **1**. A streamlined representation is shown in Figure 4. The strong current-density pathway follows the inner perimeter of the macroring, similar to the current density of molecule **1**. The ring-current strength of a molecular ring consisting of four Zn–porphyrins connected with ethyne linkers also sustains a paratropic ring current of −13.7 nA·T^–1^, whereas a ring with six Zn–porphyrins connected with ethyne linkers is almost nonaromatic, sustaining a ring current of −2.6 nA·T^–1^. Thus, larger neutral Zn–porphyrin rings are nonaromatic regardless of whether the linker is an ethyne or a butadiyne bridge.^24,32^ Integration of the current density passing through planes 2B and 2C of molecule **2** shows that there is almost no current-density flow between the joining linkers of the two macrorings. The integrated current-density strengths passing through the planes in Figure 3 are summarized in Table 2. The Zn–porphyrin moieties in Figure 3c also sustain a local diatropic ring current of 16.6 nA·T^–1^.

**Table 2:** Strength of the Diatropic and Paratropic Contributions to the Net Strength of the Current Density (in nA·T^–1^) Passing Different Integration Planes in Molecule **2**

plane	diatropic	paratropic	net
2A	2.8	–20.7	–17.9
2B	10.5	–8.1	2.4
2C	11.7	–8.6	3.1

### 3.3 Molecule **3**

**Figure 5:**
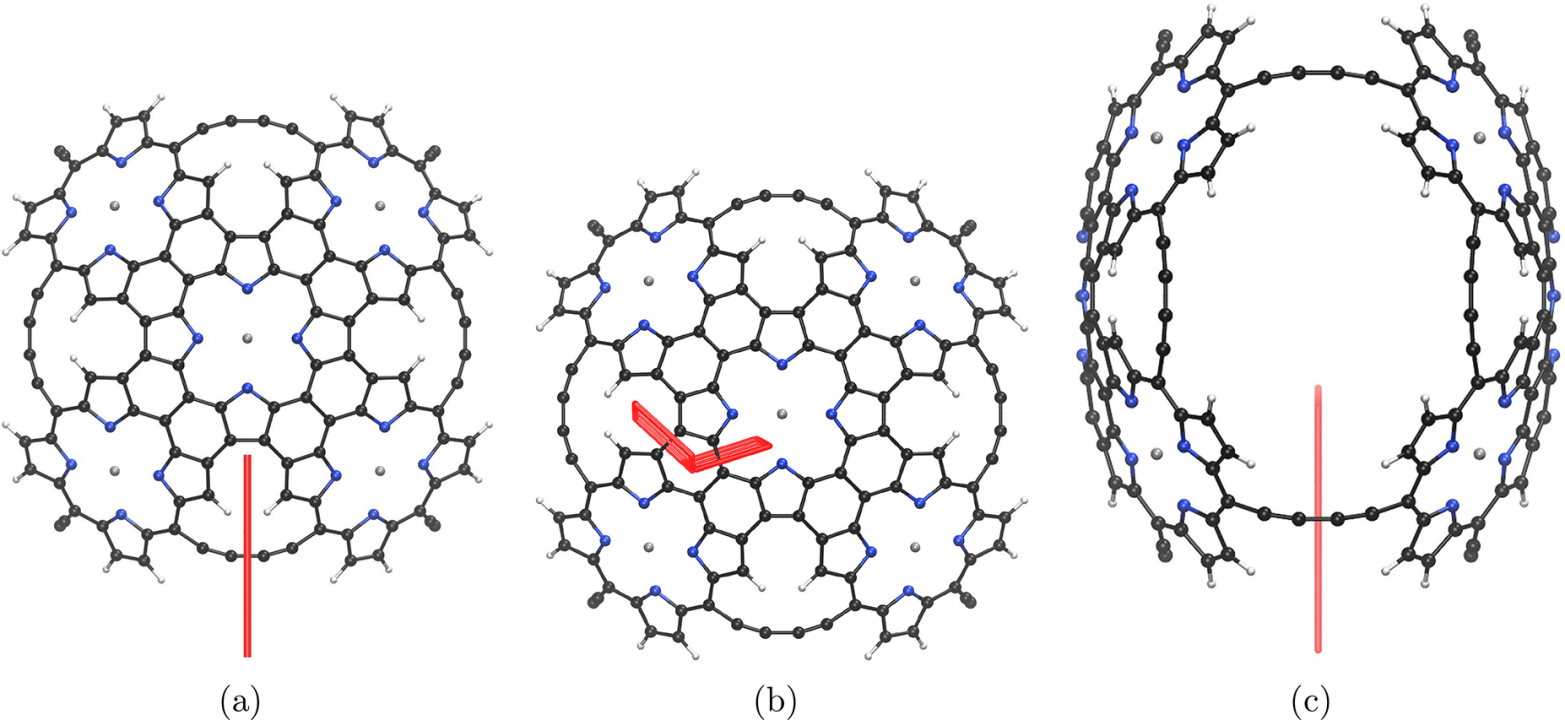
Red bars illustrate integration planes in molecule **3** used in the current density analysis. Planes 3A, 3B, and 3C are shown in parts a–c, respectively. The magnetic field points toward the viewer.

**Figure 6:**
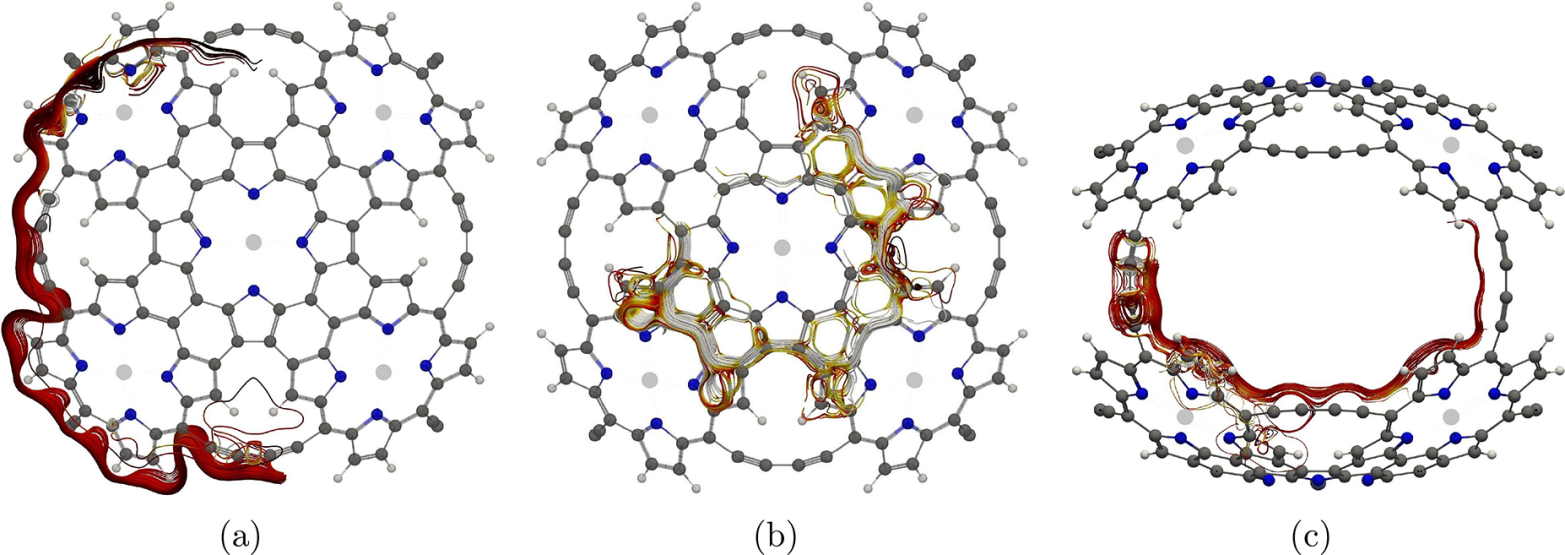
Current-density pathways in molecule **3**.

Molecule **3** resembles a clam shell in the sense that there is a pair of five directly fused Zn–porphyrin units. They are fused at the *meso*- and β-carbon atoms. The four units on the edge are connected via butadiyne linkers at the remaining *meso*-positions. Butadiyne linkers also connect the two halves of the clam-shell structure as illustrated in Figure 5 where the integration planes for the current density analysis are shown. Plane 3A is placed across the linker connecting two neighboring Zn–porphyrin units within one half of the clam shell. Plane 3B is placed at the fusion point of the Zn–porphyrin units across the C–C bond between the pyrrole ring of two neighboring Zn–porphyrin units. The strength of the current-density pathway between the two halves of the molecule was investigated by integrating the current density passing through plane 3C that cuts the linker between the two halves of the clam shell. Current-density pathways of ***3*** are shown in Figure 6. There is almost no current-density flux between the two opposite clam halves of molecule **3**. The strongest current strength is obtained when integrating the current density passing through the planes in Figure 6b when the magnetic field is perpendicular to the two halves of the clam shell. A paratropic ring current follows the fused bonds of the Zn–porphyrin units and another paratropic ring current takes the innermost route at the porphyrinoid ring. The strength of the current density passing plane 3B (left) in Figure 6b is −14.9 nA·T^–1^ and the strength of the ring current passing through plane 3B(right) is −13.2 nA·T^–1^. Another ring current can be found by tracing the outer perimeter of the molecule, i.e., the butadiyne linkers and the edge of the pyrrole rings. The strength of the current density is 10.4 nA·T^–1^, which is weaker than the sum of the paratropic ring currents. Considering both halves of molecule **3** yields a total current-density strength of −35.4 nA·T^–1^ when the magnetic field is perpendicular to the plane seen by the viewer in parts a and b of Figure 6. The strength of the current-density passing through the integration planes in Figure 5 are summarized in Table 3.

**Table 3:** Strength of the Diatropic and Paratropic Contributions to the Net Strength of the Current Density (in nA·T^–1^) Passing Different Planes of Molecule **3**

plane	diatropic	paratropic	net
3A	15.0	–4.7	10.4
3B(left)	6.0	–20.9	–14.9
3B(right)	7.0	–20.2	–13.2
3C	6.8	–8.6	–1.8

### 3.4 Molecule **4**

**Figure 7:**
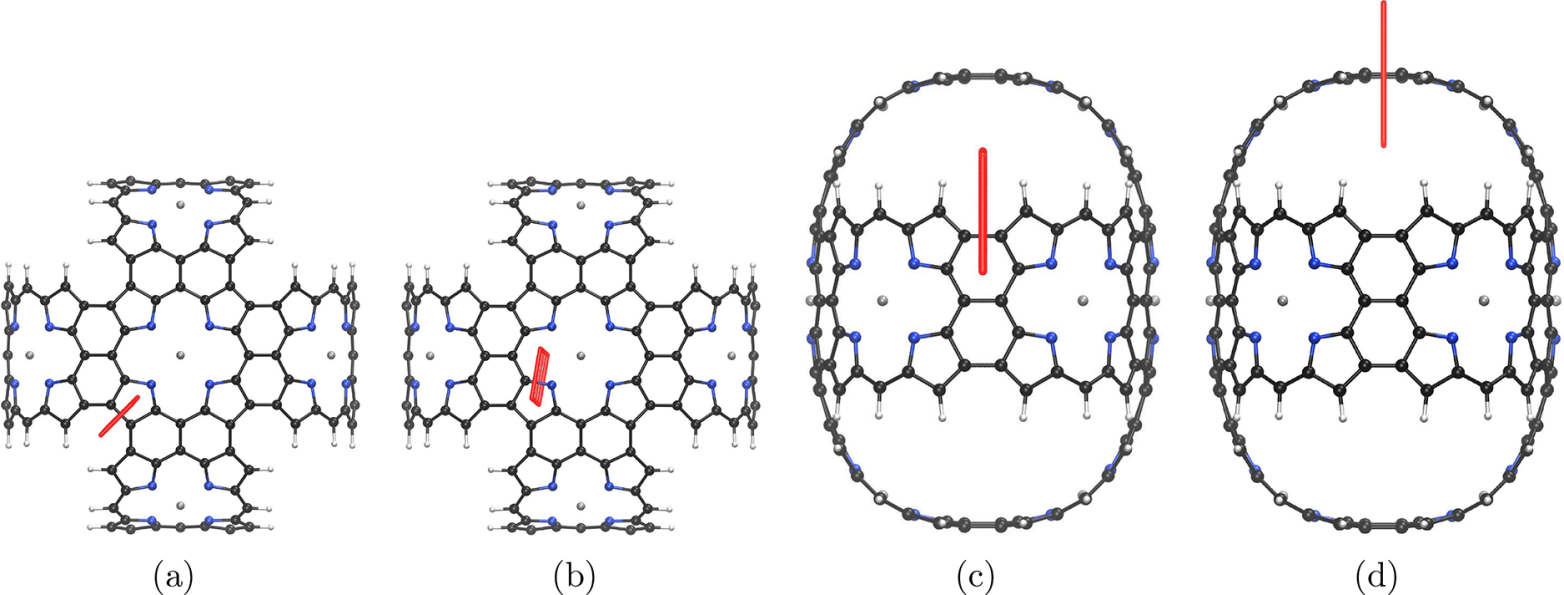
Red bars illustrate integration planes in molecule **4** used in the current density analysis. Planes 4A, 4B, 4C, and 4D are shown in parts a–d, respectively. The magnetic field points toward the viewer.

**Figure 8:**
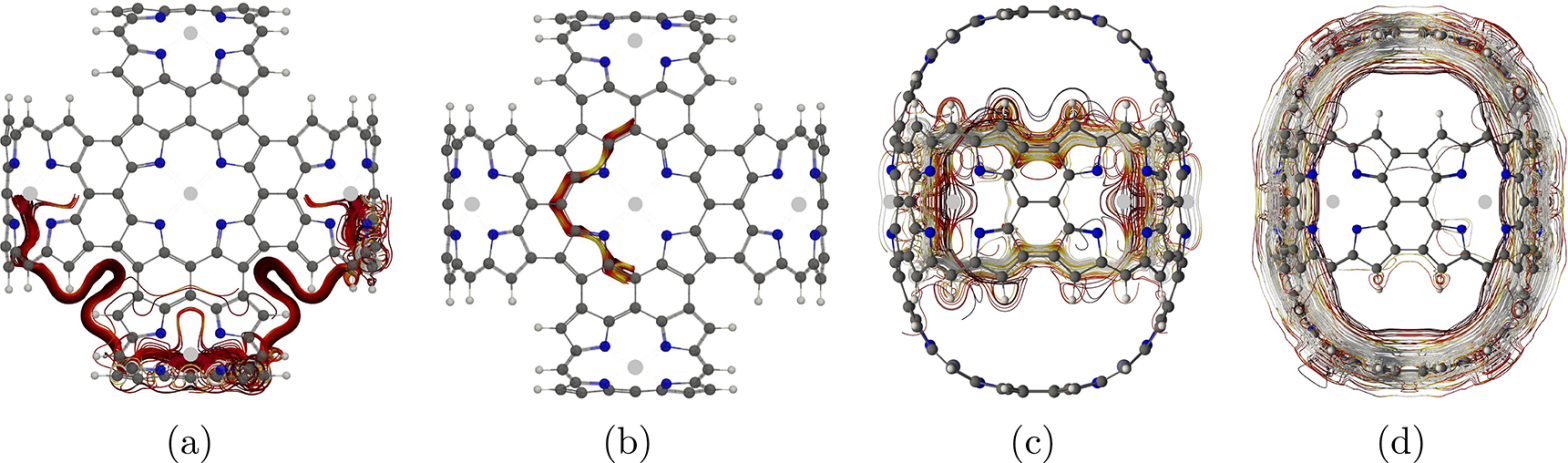
Current-density pathways in the neutral molecule **4**.

**Figure 9:**
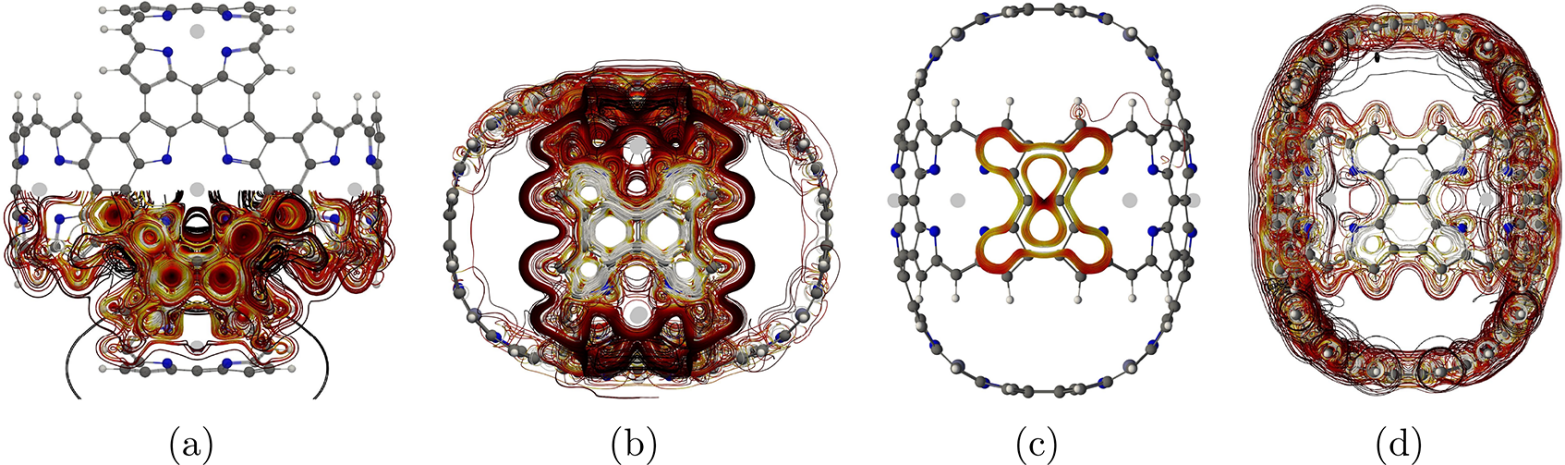
Current-density pathways in the dication of molecule **4** are shown.

Molecule **4** consists of 10 Zn–porphyrin units forming two crossed macrorings, each comprising six Zn–porphyrin units that are directly fused at the *meso* carbons; i.e., there are no linkers. Two of the Zn–porphyrin units are shared by the two macrorings. The molecule is analogous to the recently synthesized porphyrinoid structures consisting of two intersecting macrorings.^11^ The current density was analyzed by employing the integration planes shown in Figure 7. Molecule **4** was investigated both as a neutral species and as a dication because in other studies, the aromatic character of the Zn–porphyrin macrorings changes from aromatic to antiaromatic or vice versa upon oxidation. Current-density pathways of **4** and its dication are shown in Figure 8 and Figure 9, respectively.^24,32^ Plane 4A is placed across the bond between the β carbon atoms of a pyrrole ring, giving the global current-density pathway whose strength is 10.1 nA·T^–1^ in the neutral species and 14.4 nA·T^–1^ in the dication. The current density tracing the inner contour of the Zn–porphyrin unit is quantified using plane 4B placed across a C–N bond. The current strength is rather similar in both species, i.e., 6.5 nA·T^–1^ in the neutral and 7.5 nA·T^–1^ in the charged molecule, suggesting that molecule **4** and its cation are aromatic when the magnetic field is perpendicular to the planes in parts a and b of Figure 7.

Applying the magnetic field perpendicularly to the six-membered rings of molecule **4**, which are formed when fusing two neighboring Zn–porphyrin units, is shown in Figure 7c. The ring current flows then around several five- and six-membered rings in molecule **4** and its dication as shown in Figure 8c. The dication **4** also sustains two local current-density pathways as shown in parts b and c of Figure 9. One current-density pathway goes around the four pyrrole rings, and the other one is inside the two six-membered rings. These strong paratropic current-density pathways of about −30 nA·T^–1^ are compensated by a diatropic current-density flux on the outside of the fused Zn–porphyrin moieties with strengths 4.9 and 4.5 nA·T^–1^ for the neutral molecule **4** and its dication, respectively.

The strength of the global ring current of the macroring is obtained by integrating the current density passing through plane 4C in Figure 7c. The current density of molecule **4** is strongly paratropic with a strength of −212.8 nA·T^–1^. This value may be overestimated, because the B3LYP functional was employed,^26^ but still, the molecular ring is strongly antiaromatic. The large ring of the dication is nonaromatic with a vanishing net ring-current strength. The current density along the macroring consists of many local vortices as seen in Figure 9d. The strengths of the current density passing through the integration planes in Table 4 show that the current densities of molecule **4** and its dication are similar except for the current-density of the macroring, whose aromatic character changes from strongly antiaromatic in the neutral molecule **4** to nonaromatic in its dication.

**Table 4:** Strength of the Diatropic and Paratropic Contributions to the Strength of the Current Density (in nA·T^–1^) Passing Different Planes of Molecule **4**

plane	diatropic	paratropic	net
neutral			
4A	16.8	–6.7	10.1
4B	14.5	–8.0	6.5
4C	4.9	–30.3	–25.4
4D	5.2	–218.0	–212.8
dication			
4A	5.9	–20.4	14.4
4B	15.1	–7.6	7.5
4C	4.5	–32.0	–27.5
4D	19.5	–20.4	–0.9

### 3.5 Molecule **5**

**Figure 10:**
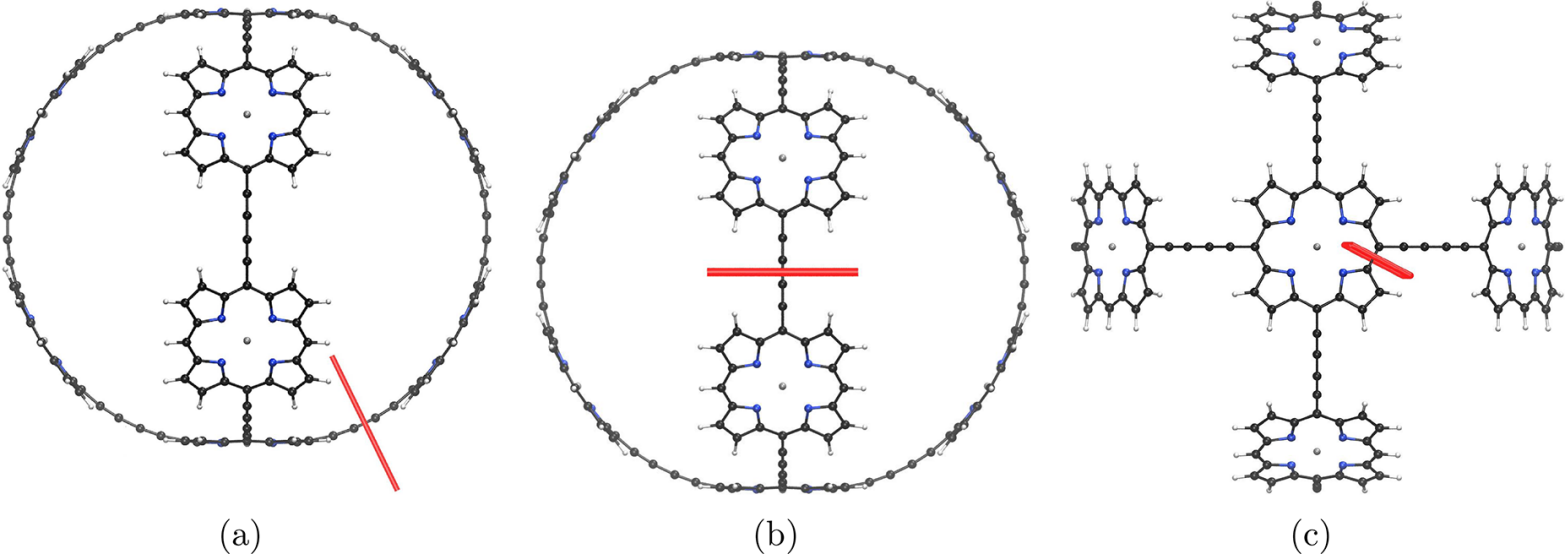
Red bars illustrate the horizontal edge of the integration planes in molecule **5** used in the current density analysis. Planes 5A, 5B, and 5C are shown in parts a–c, respectively. The magnetic field is perpendicular to the plane seen by the reader.

Molecule **5** is analogous to molecule **4**; however, in **5**, the 10 Zn–porphyrin rings are connected via butadiyne linkers. It consists of two macrorings with six Zn–porphyrin units in each ring where two of the units are common for both of the rings. Molecule **5** was investigated both as a neutral species and as a dication. The magnetically induced current density of the macroring of molecule **5** was investigated by placing an integration plane such that it crosses the current-density flux associated with the macroring as shown in Figure 10a. The strength of the current density passing through the integration planes in Figure 10 are given in Table 5. The neutral molecule is nonaromatic, sustaining a very weak ring-current of −2.4 nA·T^–1^, whereas the dication of **5** is strongly aromatic, sustaining a net ring-current of 34.6 nA·T^–1^. A similar alternating aromatic character was obtained for Zn–porphyrin nanorings in the study by Peeks et al.^24^ The integration plane crossing the linker of the other macroring shows that for the direction of the magnetic field in Figure 10b, only local current-density pathways pass through that plane, yielding a vanishing global ring-current strength.

**Table 5:** Strength of the Diatropic and Paratropic Contributions to the Net Strength of the Current Density (in nA·T^–1^) passing the integration planes of molecule **5** shown in Figure 10

plane	diatropic	paratropic	net
neutral			
5A	6.4	–8.9	–2.4
5B	7.6	–7.6	0.0
5C	22.3	–4.3	18.0
dication			
5A	34.7	–0.1	34.6
5B	7.7	–7.7	0.0
5C	17.8	–5.0	12.8

**Figure 11:**
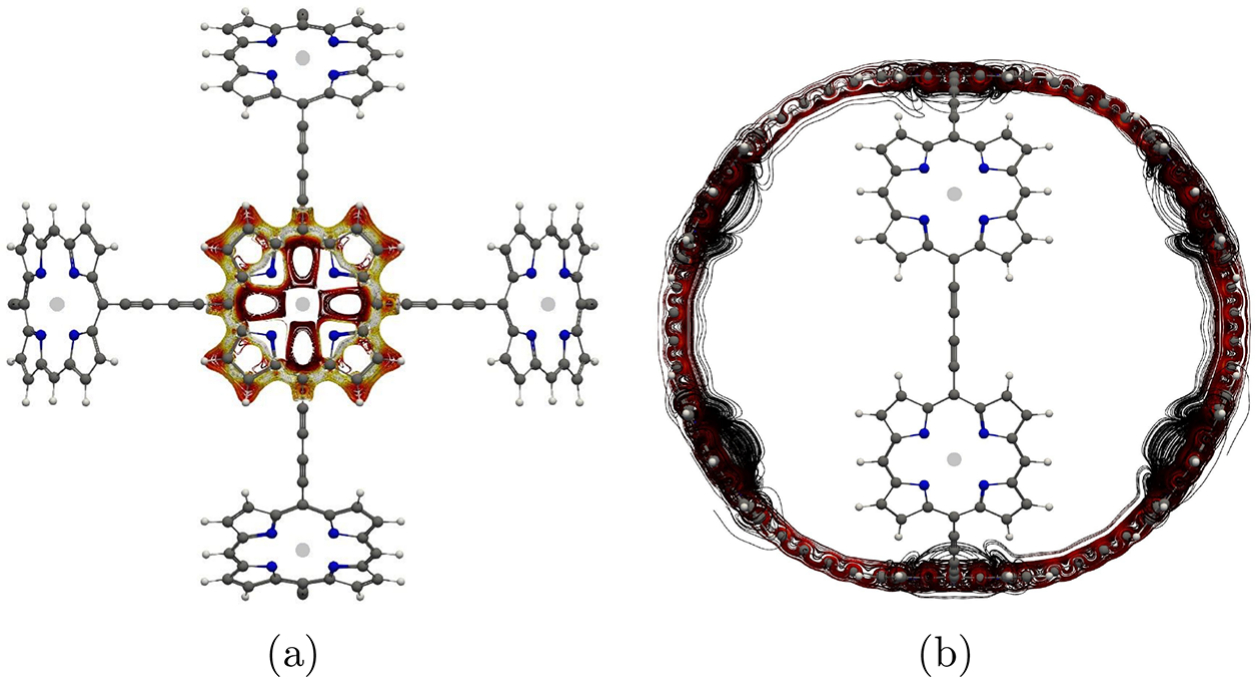
Current-density pathways in the dication of molecule **5**.

Applying the magnetic field perpendicularly to a Zn–porphyrin ring leads to local aromaticity of the neutral molecule **5** and its dication. Integration of the current density shown in Figure 11a yielded ring-current strengths of 18.0 and 12.8 nA·T^–1^ for molecule **5** and its dication, respectively. The current-density flux around the macroring of the dication of ***5*** is shown in Figure 11b. The aromatic character of the Zn–porphyrin moieties is, though, weaker than for free-base porphyrin, whose ring-current strength is 27.2 nA·T^–1^.^67^

## 4 Conclusions

Porphyrin chemistry has been a blooming field in the recent years, and many intriguing nanostructures have been synthesized with potentially useful properties in optoelectronics and other application fields. We have investigated the current density and the aromaticity of five nanosized molecular cages consisting of Zn–porphyrin units connected by linkers of different length. Molecule **1** is an ellipsoid with three crossing macrorings. Molecule **2** is a porphyrinoid nanotube consisting of two ethyne linked porphyrinoid rings. Molecule **1** is obtained when adding two porphyrinoid rings at the ends of molecule **2**. Molecule **3** consist of two fused Zn–porphyrins shells that are connected via butadiyne bridges. Molecule **4** consists of two crossing fused Zn–porphyrin belts. Molecule **5** consists of two crossing Zn–porphyrin rings connected with butadiyne bridges. It is structurally similar to the one synthesized by Anderson et al. but smaller.^11^ The macrorings consisting of C_2_- or C_4_-linked porphyrinoids are antiaromatic or nonaromatic since the rings comprise of an even number Zn–porphyrins that has 26 π electrons each. The C_2_- or C_4_-linked Zn–porphyrin moieties perpendicular to the applied magnetic field are generally aromatic.

The structures were constructed from polyhedral graphs with degree-4 vertices. Due to the size of the molecules, the structure optimizations were done stepwise. The final structures were optimized at the density functional theory (DFT) level using the BP86 functional, the def2-SVP basis set and the D3(BJ) dispersion correction, which is a commonly used level of theory for ordinary molecules.

The nuclear magnetic shielding tensors were calculated at the B3LYP/def2-SVP level. The current densities were calculated at the same level using the gauge-including magnetically induced current (GIMIC) method.^57−60^ The current densities were used for determining the aromatic character, the degree of aromaticity and the current-density pathways. Visual analysis of the current-density field was used to identify current-density pathways and to determining how to place integration planes for assessing the strength of the current-density flux along the main current-density pathways. The neutral species of four of the five molecules sustain strong paratropic ring currents, suggesting that they are antiaromatic, because there are 4*n* electrons in the conjugation pathway.

Molecules **1** and **2** sustain a paratropic ring current around the nanotube when the magnetic field is directed longitudinally, whereas they do not sustain any strong ring currents when the magnetic field is perpendicular to the four-unit macroring. The Zn–porphyrin units sustain a local ring current when the magnetic field is perpendicular to them. However, the local ring current of the Zn–porphyrins is much weaker than for Zn–porphyrin, whose ring-current strength is 26.7 nA/T. Molecule **5** also sustains a similar local ring current in the Zn–porphyrin unit, when the magnetic field is perpendicular to it. The local ring-current strengths in the Zn–porphyrin units are in the range 12.0–18.0 nA·T^–1^ for molecules **1**, **2**, and **5** and the dication of molecule **5**.

Fusing porphyrinoids as in molecules **3** and **4** results in complicated current-density pathways as previously observed for fused porphyrinoid belts.^31^ The current-density pathways significantly differ from the ones usually appearing in porphyrinoids and butadiyne linked porphyrinoids. The two halves of molecule **3** are globally antiaromatic, whereas there is no current-density flux between them.

Molecules **4** and **5** are two crossing macrorings. The aromatic character of the main molecular ring of molecules **4** and **5** changes upon oxidation. The neutral form of molecule **5** is nonaromatic, whereas a very strong paratropic ring current of −212.8 nA·T^–1^ is sustained by the macroring of molecule **4**. The current-density strength of molecule **4** might be overestimated at the B3LYP level. However, it is certain that it sustains a paratropic ring current, making the molecule strongly antiaromatic. The current density of the dication of molecule **4** forms local vortices in the molecular rings leading to nonaromatic character. The neutral form of molecule **5** is nonaromatic, whereas its cation exhibits a strong aromatic character with a global ring-current strength of 34.6 nA·T^–1^. This kind of alternating aromatic character upon oxidation is common in porphyrinoid nanostructures.^10,11,24,30,32,34,37−39^

## Supporting Information Available

Cartesian coordinates (in Å) of the molecular structures (ZIP)
